# Supplementary material for: ABA-independent PP2C-binding in PYLs traces to bacterial origins and persists in land plants
Source: Nat Commun. 2025 Dec 16;16:11261. doi: 10.1038/s41467-025-66141-9 (PMC12717121; doi:10.1038/s41467-025-66141-9)
Supplement: Supplementary file 1 — Supplementary Information [file 41467_2025_66141_MOESM1_ESM.pdf]

ABA-independent PP2C-binding in PYLs traces to bacterial origins and persists in land plants

Tianjiao Lu[1,2#], Qingzhong Li[1#], Tao Hu[1,3], Wenqi Li[4], Yafei Lu[4], Huiling Huang[1,2], Yang Zhao[1,2*]

[1]Key Laboratory of Plant Carbon Capture, Shanghai Center for Plant Stress Biology, CAS Center of Excellence in Molecular Plant Sciences, Chinese Academy of Sciences, Shanghai 200032, China

[2]University of Chinese Academy of Sciences, Beijing 100049, China

[3]State Key Laboratory of Herbage Improvement and Grassland Agro-ecosystems, College of Pastoral Agriculture Science and Technology, Lanzhou University, Lanzhou 730020, China

[4]State Key Laboratory of Bio-membrane and Membrane Biotechnology, Center for Structural Biology, School of Medicine and School of Life Sciences, Tsinghua-Peking Center for Life Sciences, Tsinghua University, Beijing 100084, China

# These authors contributed equally.

*e-mail: yangzhao@psc.ac.cn.

Supplementary materials

Contains:

Description of Supplementary Data 1

Supplementary Figure 1-5

Supplementary Table 1

Supplementary References

Description of Additional Supplementary Files:

Supplementary Data 1: Phylogenetic analysis of PYLs from the representative land plants and streptophyte algae.

Phylogenetic analysis of PYLs from the genome and transcriptome data of representative land plants and streptophyte algae. The two critical ABA-binding residues are listed. A third residue represents a conserved serine residue that also determines the ABA responsiveness of PYLs[3].

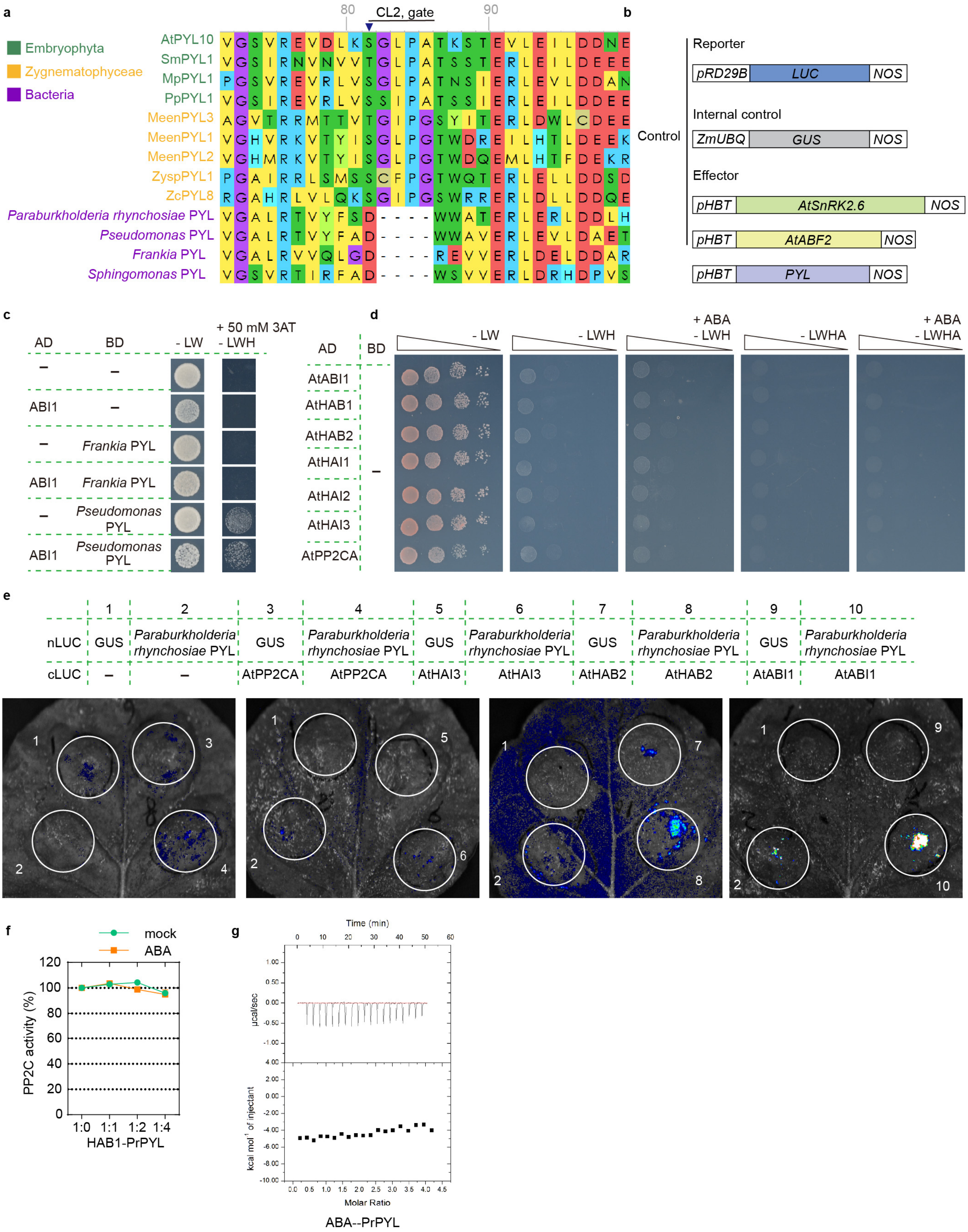

Supplementary Fig. 1 | The bacterial PYL-homolog from *Paraburkholderia rhynchosiae* carries PP2C-binding ability.

a Sequence alignment of PYL-homologs from bacteria (purple), Zygnematophyceae algae (yellow),

and embryophytes (green). The CL2 region essential for PP2C binding is marked in black, and the critical Ser/Thr residue is indicated with a blue triangle. *Meen*, *Mesotaenium endlicherianum*; *Zc*, *Zygnema circumcarinatum*; *Zysp*, *Zygnemopsis* spp. **b** Schematic representation of the reporter, effector, and internal control constructs used in the transient expression assay in protoplasts of the *Arabidopsis pyl* duodecuple mutant. *pRD29B*, *Response to Desiccation* (*RD*) *29B* gene promoter, induced by ABA and osmotic stress. *LUC*, luciferase. *ZmUBQ*, the maize ubiquitin promoter. *GUS*, β-glucuronidase. pHBT constructs are driven by the *35S* CaMV promoters. *NOS* is the terminator signal of the nopaline synthase gene. **c** Interactions between the protein phosphatase AtABI1 and PYL-homologs from bacteria in yeast two-hybrid (Y2H) assay. The PYL-homologs from *Frankia* and *Pseudomonas* were used. Yeast cells were grown on the nonselective SD/−Leu−Trp (−LW) medium and the selective SD/−Leu−Trp−His (−LWH) medium with 50 mM 3-Amino-1,2,4-triazole (3-AT) to reduce the background growth. AD-ABI1/BD and AD/BD-PYLs served as negative controls. **d** Negative controls for interactions between *Arabidopsis* clade A PP2Cs and *Paraburkholderia rhynchosiae* PYL (PrPYL) in Y2H assay in Fig. 1d. Yeast cells were grown on the nonselective SD/−LW and the selective SD/−LWH or SD/−LWHA, without or with 10 μM ABA. Dilutions (10^{-1}, 10^{-2}, and 10^{-3}) of equal density were spotted. AD-PP2Cs and BD were used as negative controls. **e** Interactions between PrPYL and *Arabidopsis* clade A PP2Cs, including PP2CA, HAI3, HAB2, and ABI1, in *N. benthamiana* leaves using Firefly Luciferase Complementation Imaging (LCI) assay. GUS-nLUC was used as a negative control. **f** In PP2C enzyme activity assays with pNPP substrate, PrPYL failed to suppress HAB1 phosphatase activity in the absence or presence of 10 μM ABA. Error bars, SD (n = 3 independent repeats). **g** ITC analyses detected no binding between PrPYL and ABA. Source data are provided as a Source Data file.

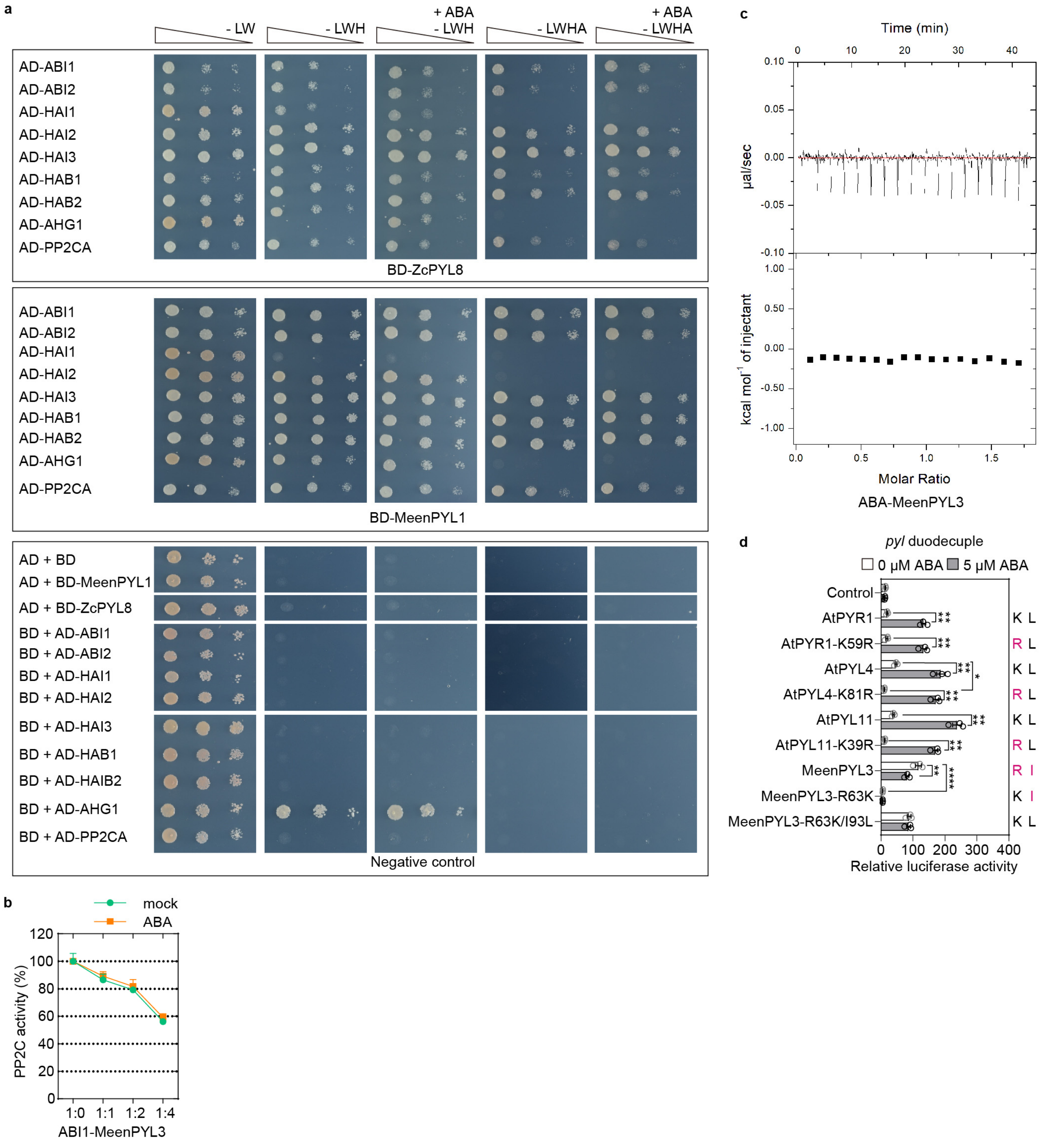

Supplementary Fig. 2 | Some PYL-homologs from Zygnematales have PP2C inhibitory activity. **a** Interactions between *Arabidopsis* clade A PP2Cs and PYL-homologs from Zygnematophyceae algae, including ZcPYL8 (from *Zygnema circumcarinatum*) and MeenPYL1 (from *Mesotaenium endlicherianum*), in Y2H assay. The transformed yeast cells were grown on the nonselective SD/−LW and the selective SD/−LWH or SD/−LWHA media, without or with 10 μM ABA. Zygnematophyceae-derived-PYLs were fused with the GAL4 DNA-binding domain (BD), and the clade A PP2Cs were

fused with the GAL4 activation domain (AD). Dilutions (10^{-1} and 10^{-2}) of equal density were spotted. The combinations of AD/BD, AD/BD-PYLs and AD-PP2Cs/BD served as negative controls. **b** In PP2C enzyme assays with pNPP substrate, MeenPYL3 suppresses ABI1 phosphatase activity regardless of 10 µM ABA. Error bars, SD (n = 3 biological repeats). **c** ITC analyses demonstrate no detectable binding between MeenPYL3 and ABA. **d** The impact of alteration of key ABA carboxyl group-binding sites on properties of AtPYLs and MeenPYL3 showing that arginine is better than lysine in CL1 to generate PYL ABA receptors with no basal activity. ABA-dependence of wild-type and mutated PYLs was evaluated by *RD29Bpro:LUC* expression in *pyl* duodecuple mutant protoplasts transformed with *PYLs*. Transformation with *SnRK2.6*, *ABF2* and *RD29Bpro:LUC* served as control. PYL homologs from land plants, including AtPYR1, AtPYL4, and AtPYL11, were used as positive controls for ABA receptors. Error bars, SEM (n = 3 independent biological repeats). *$p < 0.05$, ***$p < 0.001$, ****$p < 0.0001$, two-way ANOVA. Right panel: two residues critical for ABA binding in *Arabidopsis* are labeled in black, while the corresponding variant residues are labeled in red. Exact *p* values are provided in Source Data.

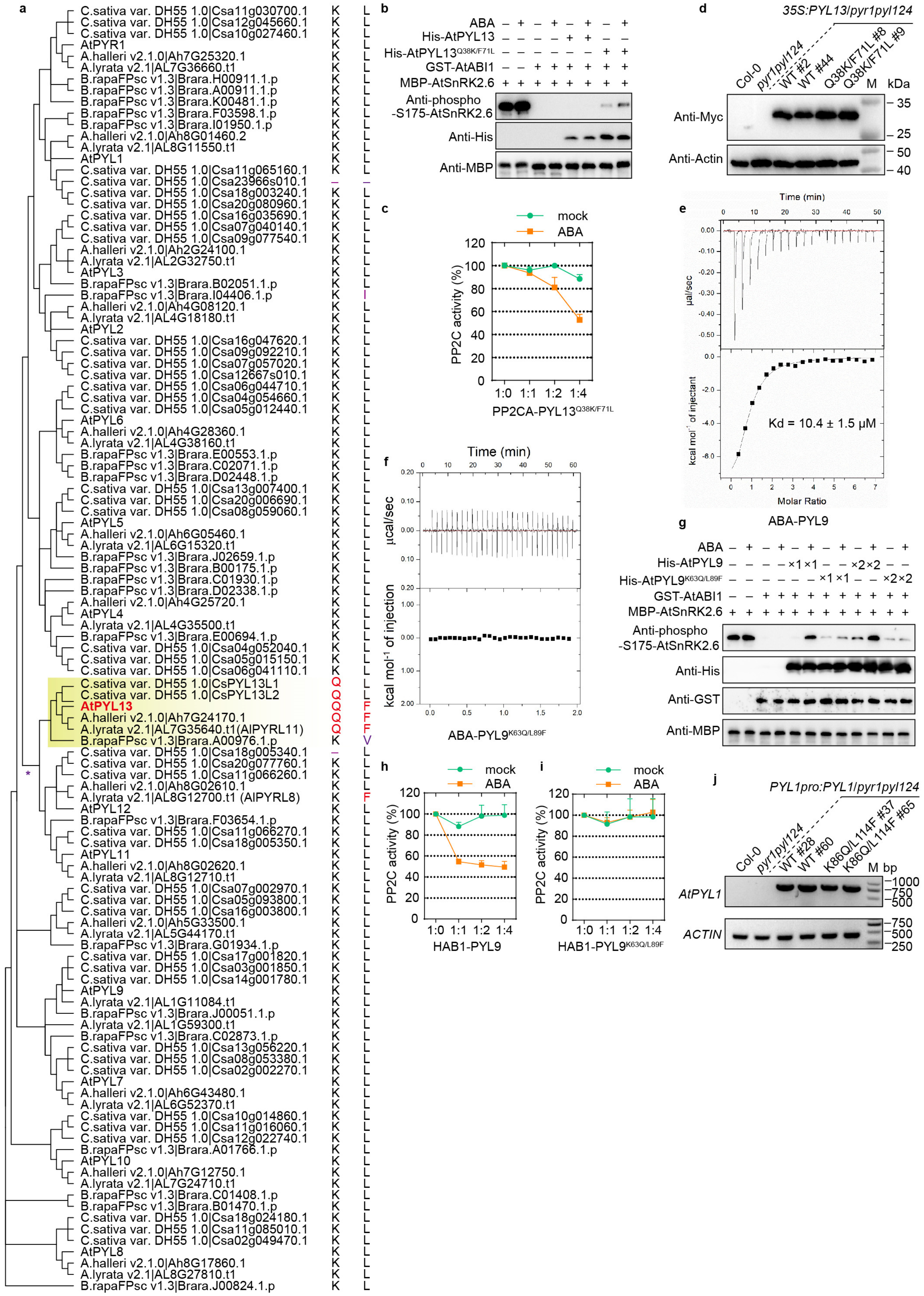
a
C.sativa var. DH55 1.0|Csa11g030700.1
C.sativa var. DH55 1.0|Csa12g045660.1
C.sativa var. DH55 1.0|Csa10g027460.1
AtPYR1
A.halleri v2.1.0|Ah7G25320.1
A.lyrata v2.1|AL7G36660.t1
B.rapaFPsc v1.3|Brara.H00911.1.p
B.rapaFPsc v1.3|Brara.A00911.1.p
B.rapaFPsc v1.3|Brara.K00481.1.p
B.rapaFPsc v1.3|Brara.F03598.1.p
B.rapaFPsc v1.3|Brara.I01950.1.p
A.halleri v2.1.0|Ah8G01460.2
A.lyrata v2.1|AL8G11550.t1
AtPYL1
C.sativa var. DH55 1.0|Csa11g065160.1
C.sativa var. DH55 1.0|Csa23966s010.1
C.sativa var. DH55 1.0|Csa18g003240.1
C.sativa var. DH55 1.0|Csa20g080960.1
C.sativa var. DH55 1.0|Csa16g035690.1
C.sativa var. DH55 1.0|Csa07g040140.1
C.sativa var. DH55 1.0|Csa09g077540.1
A.halleri v2.1.0|Ah2G24100.1
A.lyrata v2.1|AL2G32750.t1
AtPYL3
B.rapaFPsc v1.3|Brara.B02051.1.p
B.rapaFPsc v1.3|Brara.I04406.1.p
A.halleri v2.1.0|Ah4G08120.1
A.lyrata v2.1|AL4G18180.t1
AtPYL2
C.sativa var. DH55 1.0|Csa16g047620.1
C.sativa var. DH55 1.0|Csa09g092210.1
C.sativa var. DH55 1.0|Csa07g057020.1
C.sativa var. DH55 1.0|Csa12667s010.1
C.sativa var. DH55 1.0|Csa06g044710.1
C.sativa var. DH55 1.0|Csa04g054660.1
C.sativa var. DH55 1.0|Csa05g012440.1
AtPYL6
A.halleri v2.1.0|Ah4G28360.1
A.lyrata v2.1|AL4G38160.t1
B.rapaFPsc v1.3|Brara.E00553.1.p
B.rapaFPsc v1.3|Brara.C02071.1.p
B.rapaFPsc v1.3|Brara.D02448.1.p
C.sativa var. DH55 1.0|Csa13g007400.1
C.sativa var. DH55 1.0|Csa20g006690.1
C.sativa var. DH55 1.0|Csa08g059060.1
AtPYL5
A.halleri v2.1.0|Ah6G05460.1
A.lyrata v2.1|AL6G15320.t1
B.rapaFPsc v1.3|Brara.J02659.1.p
B.rapaFPsc v1.3|Brara.B00175.1.p
B.rapaFPsc v1.3|Brara.C01930.1.p
B.rapaFPsc v1.3|Brara.D02338.1.p
A.halleri v2.1.0|Ah4G25720.1
AtPYL4
A.lyrata v2.1|AL4G35500.t1
B.rapaFPsc v1.3|Brara.E00694.1.p
C.sativa var. DH55 1.0|Csa04g052040.1
C.sativa var. DH55 1.0|Csa05g015150.1
C.sativa var. DH55 1.0|Csa06g041110.1
C.sativa var. DH55 1.0|CsPYL13L1
C.sativa var. DH55 1.0|CsPYL13L2
AtPYL13
A.halleri v2.1.0|Ah7G24170.1
A.lyrata v2.1|AL7G35640.t1(AlPYRL11)
B.rapaFPsc v1.3|Brara.A00976.1.p
C.sativa var. DH55 1.0|Csa18g005340.1
C.sativa var. DH55 1.0|Csa20g077760.1
C.sativa var. DH55 1.0|Csa11g066260.1
A.halleri v2.1.0|Ah8G02610.1
A.lyrata v2.1|AL8G12700.t1 (AlPYRL8)
AtPYL12
B.rapaFPsc v1.3|Brara.F03654.1.p
C.sativa var. DH55 1.0|Csa11g066270.1
C.sativa var. DH55 1.0|Csa18g005350.1
AtPYL11
A.halleri v2.1.0|Ah8G02620.1
A.lyrata v2.1|AL8G12710.t1
C.sativa var. DH55 1.0|Csa07g002970.1
C.sativa var. DH55 1.0|Csa05g093800.1
C.sativa var. DH55 1.0|Csa16g003800.1
A.halleri v2.1.0|Ah5G33500.1
A.lyrata v2.1|AL5G44170.t1
B.rapaFPsc v1.3|Brara.G01934.1.p
C.sativa var. DH55 1.0|Csa17g001820.1
C.sativa var. DH55 1.0|Csa03g001850.1
C.sativa var. DH55 1.0|Csa14g001780.1
AtPYL9
A.lyrata v2.1|AL1G11084.t1
B.rapaFPsc v1.3|Brara.J00051.1.p
A.lyrata v2.1|AL1G59300.t1
B.rapaFPsc v1.3|Brara.C02873.1.p
C.sativa var. DH55 1.0|Csa13g056220.1
C.sativa var. DH55 1.0|Csa08g053380.1
C.sativa var. DH55 1.0|Csa02g002270.1
AtPYL7
A.halleri v2.1.0|Ah6G43480.1
A.lyrata v2.1|AL6G52370.t1
C.sativa var. DH55 1.0|Csa10g014860.1
C.sativa var. DH55 1.0|Csa11g016060.1
C.sativa var. DH55 1.0|Csa12g022740.1
B.rapaFPsc v1.3|Brara.A01766.1.p
AtPYL10
A.halleri v2.1.0|Ah7G12750.1
A.lyrata v2.1|AL7G24710.t1
B.rapaFPsc v1.3|Brara.C01408.1.p
B.rapaFPsc v1.3|Brara.B01470.1.p
C.sativa var. DH55 1.0|Csa18g024180.1
C.sativa var. DH55 1.0|Csa11g085010.1
C.sativa var. DH55 1.0|Csa02g049470.1
AtPYL8
A.halleri v2.1.0|Ah8G17860.1
A.lyrata v2.1|AL8G27810.t1
B.rapaFPsc v1.3|Brara.J00824.1.p
b
ABA
His-AtPYL13
His-AtPYL13Q38K/F71L
GST-AtABI1
MBP-AtSnRK2.6
Anti-phospho -S175-AtSnRK2.6
Anti-His
Anti-MBP
c
mock
ABA
PP2C activity (%)
1:0 1:1 1:2 1:4
PP2CA-PYL13Q38K/F71L
d
35S:PYL13/pyr1pyl124
Col-0
pyr1pyl124
WT #2
WT #44
Q38K/F71L #8
Q38K/F71L #9
M
kDa
Anti-Myc
Anti-Actin
35
25
50
40
e
Time (min)
µal/sec
kcal mol-1 of injectant
Kd = 10.4 ± 1.5 µM
Molar Ratio
ABA-PYL9
f
Time (min)
µcal/sec
kcal mol-1 of injection
ABA-PYL9K63Q/L89F
g
ABA
His-AtPYL9
His-AtPYL9K63Q/L89F
GST-AtABI1
MBP-AtSnRK2.6
Anti-phospho -S175-AtSnRK2.6
Anti-His
Anti-GST
Anti-MBP
h
mock
ABA
PP2C activity (%)
1:0 1:1 1:2 1:4
HAB1-PYL9
i
mock
ABA
PP2C activity (%)
1:0 1:1 1:2 1:4
HAB1-PYL9K63Q/L89F
j
PYL1pro:PYL1/pyr1pyl124
Col-0
pyr1pyl124
WT #28
WT #60
K86Q/L114F #37
K86Q/L114F #65
M
bp
AtPYL1
ACTIN
1000
750
500
250

Supplementary Fig. 3 | Two key residues are crucial for the ABA dependence of AtPYL13 and AtPYL9.

a Phylogenetic analysis of the PYLs in the Brassicaceae family using the Maximum Likelihood method. A bootstrap consensus tree was generated based on the functional annotations of PYL genes from *Arabidopsis thaliana*, *Arabidopsis lyrata*, *Arabidopsis halleri*, *Brassica rapa*, and *Camelina sativa*. Two critical ABA-binding residues are listed on the right. **b** ABA-dependence of wild-type and mutated AtPYL13 in releasing SnRK2.6 from PP2C inhibition. SnRK2 phosphorylation was detected with anti-phospho-S175-SnRK2s antibodies. SnRK2.6 activity was repressed by GST-ABI1, and PYLs released this inhibition. Protein loading was detected by anti-His antibody for His-AtPYLs and anti-MBP antibody for MBP-SnRK2.6. **c** In PP2C enzyme assays with pNPP substrate, AtPYL13-Q38K/F71L suppresses PP2CA phosphatase activity in the presence of 10 μM ABA. Error bars, SD (n = 3 independent repeats). **d** Immunoblotting results using anti-Myc antibody showing the protein abundance of wild-type and mutated AtPYL13 in transgenic lines driven by the constitutive *35S* cauliflower mosaic virus (CaMV) promoter in the *pyr1pyl124* quadruple mutant background. ACTIN served as loading control. **e, f** ITC analyses demonstrate no detectable binding between AtPYL9-K63Q/L89F and ABA. **g** ABA-dependence of wild-type and K63Q/L89F-mutated AtPYL9 in regulating SnRK2.6 release from ABI1 inhibition. SnRK2 phosphorylation was detected with anti-phospho-S175-SnRK2s antibodies. Protein loading was detected by anti-His antibody for His-AtPYLs, anti-GST antibody for GST-ABI1 and anti-MBP antibody for MBP-SnRK2.6. **h, i** In PP2C enzyme assays with pNPP substrate, AtPYL9, but not AtPYL9-K63Q/L89F, suppresses HAB1 phosphatase activity in the presence of 10 μM ABA. Error bars, SD (n = 3 independent repeats). **j** The expression level of wild-type and mutated *AtPYL1* driven by the *AtPYL1* native promoter in the *pyr1pyl124* quadruple mutant background. The gene expression level was analyzed by RT-PCR using the paired PYL1-F and HA-R primers, which are listed in the Primer table. *ACTIN3* was used as an internal reference. Source data are provided as a Source Data file.

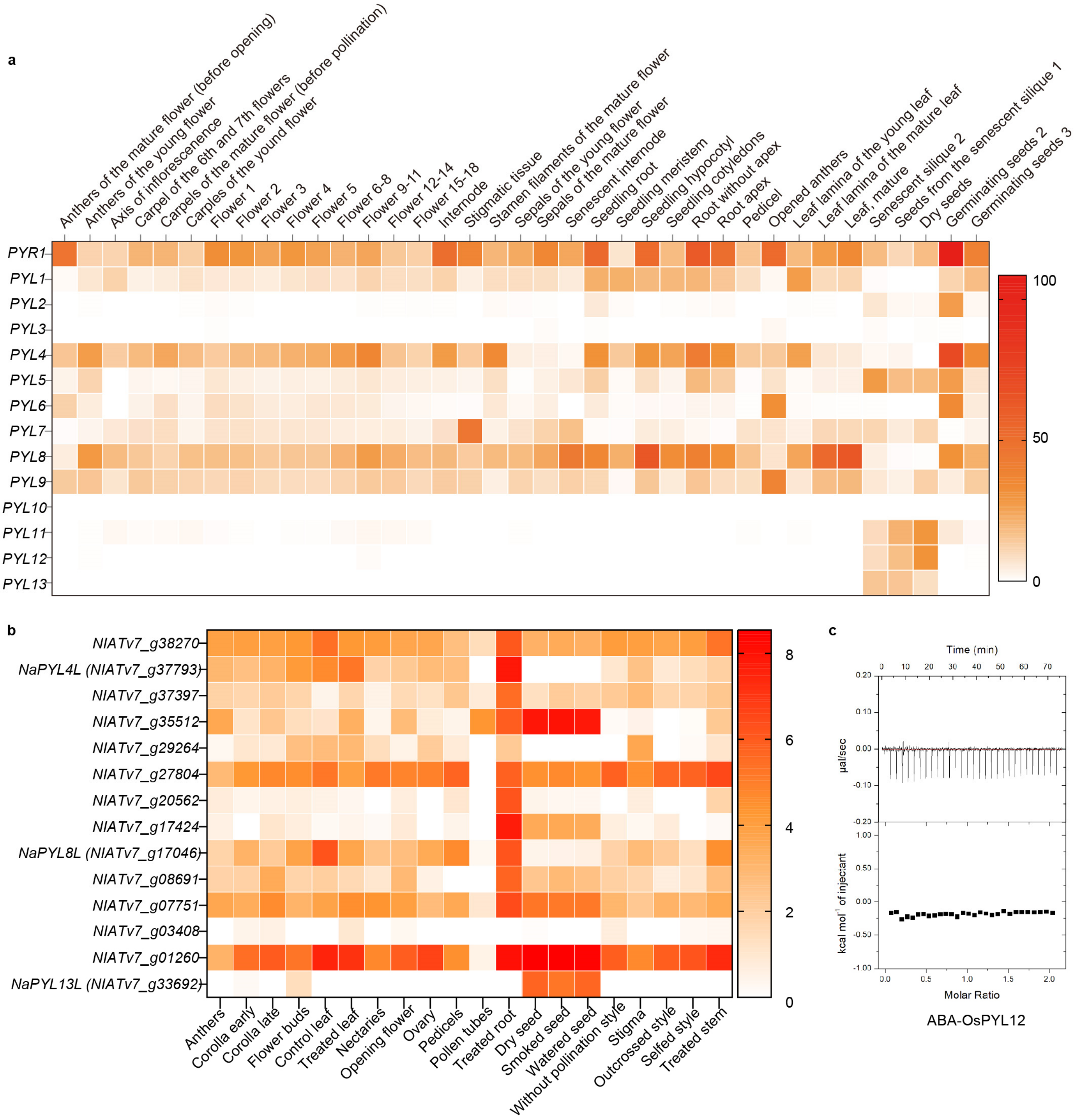

Supplementary Fig. 4 | Expression pattern of ABA-independent *PYL*-like genes in vascular plants.

a Heat map showing the expression level of *AtPYL* genes in different tissues and developmental stages of *Arabidopsis*. Data is derived from the Arabidopsis eFP browser. **b** Heat map showing the expression level of *PYL* genes in seeds and other tissues of *Nicotiana attenuata*. Data is derived from the eFP browser on *Nicotiana attenuata* Data Hub (http://nadh.ice.mpg.de/NaDH/). **c** ITC analyses demonstrate no detectable binding between OsPYL12 and ABA. Source data are provided as a Source

Data file.

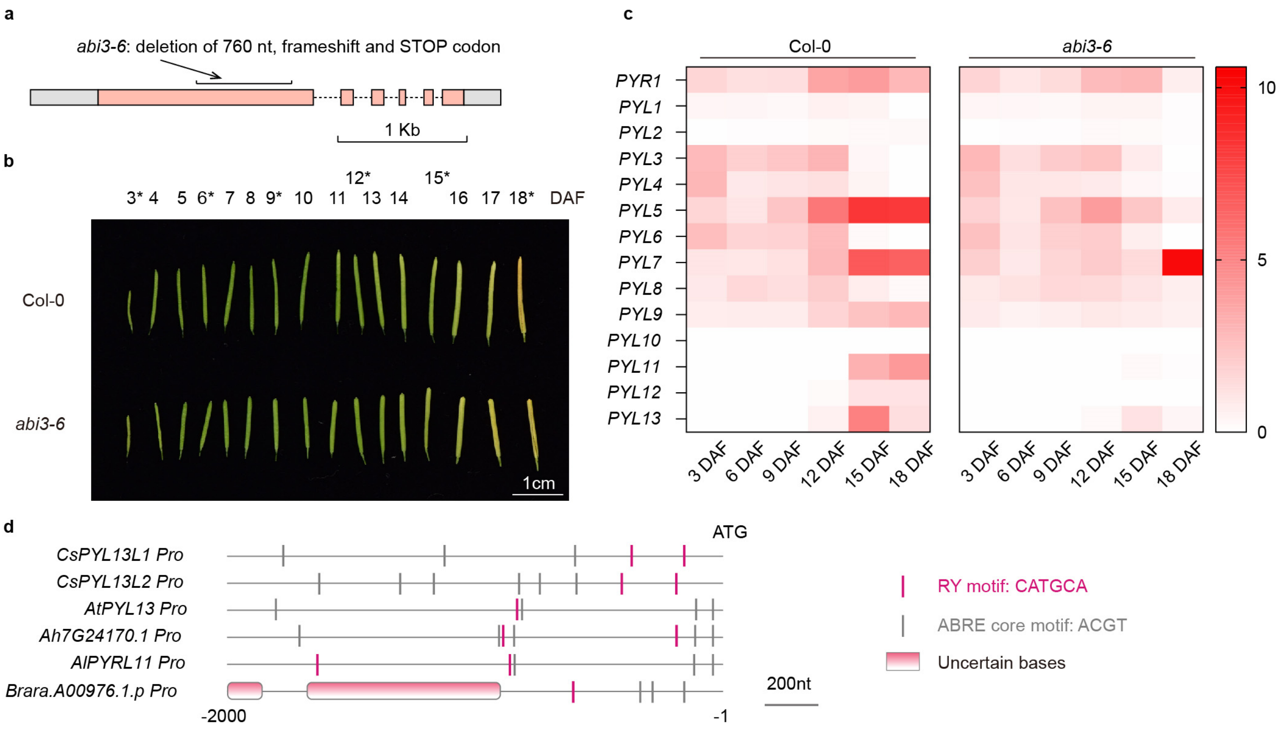

Supplementary Fig. 5 | Seed-specific expression of *AtPYL13* is mediated by ABI3 in *Arabidopsis*.

a Schematic structure of the *ABI3* gene. The mutation of *abi3-6* is indicated. UTRs are shown in gray, while exons are shown in pink. The introns are shown as dashed lines. The black line segment represents deletion in *abi3-6*. **b** Silique morphology of Col-0 wild type and *abi3-6* mutant at different sampling time points. DAF, days after flowering. Specific time points for sampling were labeled with black asterisks for the *PYL* expression assay. Scale bars, 1 cm. **c** Heat map showing the relative expression levels of *AtPYL* genes at different time points during seed development and maturation. *PP2AA3* was utilized as an internal reference. **d** Schematic illustration of candidate ABI3 binding motifs in promoters of *AtPYL13*-like genes *in* Brassicaceae family. Source data are provided as a Source Data file.

Supplementary Table 1: Primers used in this study

Experiment	Name	Sequence
Site-directed mutagenesis	*PYR1-K59Q*	F: CAAACCACAAACATACCAACACTTCATCAAATCCTG
		R: GATTTGATGAAGTGTTGGTATGTTTGTGGTTTGT
	PYR1-L87F	F: GTCATCAGTGGATTCCCGGCGAACACATCAACGGAAA
		R: GATGTGTTCGCCGGGAATCCACTGATGACGA
	PYL1-K86Q	F: TAGGCCACAGATTTACCAACACTTCATCAAAAGCT
		R: CTTTTGATGAAGTGTTGGTAAATCTGTGGCCTATCG
	PYL1-L114F	F: ACGTGATAAGTGGATTCCCGGCGAATACGTCTCGA
		R: CGTATTCGCCGGGAATCCACTTATCACGTTCACG
	PYL2-K64Q	F: AACCCCGAACGCTACCAACACTTTGTAAAAAGGTGCCG
		R: CACCTTTTTACAAAGTGTTGGTAGCGTTCGGGGTTGTC
	PYL2-L91F	F: TGACCGTAATCTCCGGCTTCCCAGCCTCAACCAGTAC
		R: GTTGAGGCTGGGAAGCCGGAGATTACGGTCAC
	PYL3-K79Q	F: CCAATCCAAACAAATACCAACACTTCATCAAG
		R: CTCTTGATGAAGTGTTGGTATTTGTTTGGAT
	PYL3-L111F	F: CGTGGTCTCTGGTTTTCCAGCGTCAACAAGCG
		R: TTGACGCTGGAAAACCAGAGACCACGCTAAC
	PYL4-K81Q	F: AACCCACAAGCTTACCAACACTTTCTCAAAAGCTGTAGCG
		R: ACAGCTTTTGAGAAAGTGTTGGTAAGCTTGTGGGTTA
	PYL4-L109F	F: CCACGTCGTCTCTGGTTTCCCCGCCGCTAGCTCCACCGAGA
		R: GGAGCTAGCGGCGGGGAAACCAGAGACGACGTGGACTTG
	PYL5-K87Q	F: CCGAAGGTTTAC C AGAACTTCATCAGACAGTGCCGTATCG
		R: GATGAAGTTCT G GTAAACCTTCGGATTATCGAAACG
	PYL5-L115F	F: TGGTCTCTGGA T TCCCGGCGGTCTCGAGCACCGAGA
		R: CGCCGGGA A TCCAGAGACCACCATGACCTCCCGGAG
	PYL6-K90Q	F: CTCAAGCGTAC C AACACTTCGTGAAAAGCTGCCACGTGGT
		R: CGAAGTGTT G GTACGCTTGAGGGTGTTCGAAGCGGCTTA
	PYL6-L118F	F: GTCTCTGGT T TCCCCGCGGCGTTTAGCTTAGAGCGG
		R: CGCGGGGA A ACCAGAGACGACTCTGACCTCTCTCAC
	PYL7-K65Q	F: GCAGAAATAC C AACCATTTATAAGCAGATGCACTGT
		R: CTTATAAATGGTT G GTATTTCTGCGGCTGATCAAATCT
	PYL7-L91F	F: CAAATCTGGT T TTCCAGCAACCACCAGTACAGAGAG
		R: TTGCTGGAA A ACCAGATTTGACATTTACTTCTCTGAGAC
	PYL8-K61Q	F: CACAGAAGTAT C AGCCGTTTATCAGTAGATGTGTGG
		R: TAAACGGCT G ATACTTCTGTGGCTGATCAAATCTTC
	PYL8-L87F	F: GAAATCTGGA T TACCAGCAACTAGAAGCACTGAGAGA
		R: TTGCTGGTA A TCCAGATTTCACATCAACTTCTCTTACTGT
	PYL10-K56Q	F: CACAAAAATAC C AACCATTTATCAGTAGGTGTGTGG
		R: TGATAAATGGTT G GTATTTTTGTGGTTCATCGAATCTCC
	PYL10-L83F	F: GAAATCTGGA C TACCAGCTACTAAAAGCACTGAG
		R: GTAGCTGGTA G TCCAGATTTCAAATCCACTTCTCTTA

	PYL12-K39Q	F: CCAAAAACATTT C AACATTTCGTAAAAACTTGTAAACTAC
		R: TACGAAATGTT G AAATGTTTTTGGGTTATCAAATC
	PYL12-L66F	F: GTTTCCGAT T TTCCGGCGAGTTTTAGCCTAGAAAGATTAGATG
		R: CGCCGGAA A ATCGGAAACCACCGTGACTTCACGG
	PYL13-Q38K	F: CCACAAGCTTATAAACGTTTCGTCAAAAGTTG
		R: TGACGAAACGTTTATAAGCTTGTGGTTTG
	PYL13-F71L	F: GTCTCCGGCTTGCCGGCGGATTTCAGCACGGA
		R: AATCCGCCGGCAAGCCGGAGACTAACGTC
	PYR1-K59R	F: CCACAAACATACAGACACTTCATCAAATCCTGCTC
		R: TTTGATGAAGTGTCTGTATGTTTGTGGTTTGTCGAA
	PYL4-K81R	F: CACAAGCTTACagaCACTTTCTCAAAAGCTGTAG
		R: TTTGAGAAAGTGtctGTAAGCTTGTGGGTTATCAAAG
	PYL11-K39R	F: TCAAGCCTACagaCAATTCGTGAAAACGTGCAA
		R: TTTCACGAATTGtctGTAGGCTTGAGGGTTATCAAAC
	MePYL3-R63K	F: CACAGAGGTTTAAATGGGCCATAGGAAGCTGTG
		R: CTATGGCCCATTTAAACCTCTGTGGCCTGTGGAAG
	MePYL3-I93L	F: AGTGACAGGGCTTCCGGGATCTTACATCACGG
		R: GATCCCGGAAGCCCTGTCACTGTCGTCATCCT
q-RT-PCR in *Nicotiana attenuate*	*NaIF5α*	F: gtcggacgaagaacaccatt
		R: cacatcacagttgtgggagg
	NaPYL4L	F: CCGATTCCATACTCACCCGG
		R: GATGACGTGGCAGCTCTTGA
	NaPYL8L	F: GCACTGAGAGGTTGGAGCTT
		R: CTTCAGGCACGTCTACCACA
	NaPYL13L	F: tggtcttcaatgccagtcca
		R: actcccaacaccaccatcac
q-RT-PCR in *Arabidopsis thaliana*	*PP2AA3*	F: CGTTACTGCCAGCCATTGTAGAACTTG
		R: CATTGCCCATTCAGGACCAAACTCTTC
	PYR1	F: CAAATCCTGCTCCGTCGAAC
		R: CCTCCGATGATACTGAATCC
	PYL1	F: CCTCCTCCTCACCAGTAAAC
		R: GGCCTATCGAAACGTCTCAC
	PYL2	F: CCAGACCCAACCACGTGCACTTC
		R: AGCCGCTCGGTACTGGTTG
	PYL3	F: TCGAACACCCTCAAGCGTAC
		R: TGAAACTGATGACGTGGCGA
	PYL4	F: GTGTTGCTCCGCCGTTATTC
		R: AGAGACGACGTGGACTTGAC
	PYL5	F: TGCAGCCTCACGATCAGACC
		R: AAGAGCCCACACGGACTCAG
	PYL6	F: AGAGGTTGGGTCGGTGAGAG
		R: CTCTTCTTGCCGTCGGAGTC

	PYL7	F: CTCAAGGCAACACCAAAG
		R: CCATTGGAAGCGTTACAG
	PYL8	F: TGGAAGCTAACGGGATTGAG
		R: GCTGGTAGTCCAGATTTCAC
	PYL9	F: TCGAGACGGTGCAATACG
		R: GCGGCTGATCAAATCTCC
	PYL10	F: GGTGGAGAGCGAGTACATCA
		R: TTACGCTACCAACCTCCAGC
	PYL11	F: GTGGAGAGTTATGTGGTG
		R: CAACTTTAGATGAGCCACCC
	PYL12	F: GTGGAGAGTTATGTGGTG
		R: TAAGTGAGCTCCATCATC
	PYL13	F: GCGTAGTCGAGACCATTG
		R: CCTCTCCGTGCTGAAATC
RT-PCR in *Oryza sativa*	*OsPP2AA3*	F: gattccgtacgcttgttggc
		R: ttccgcctcattgtcacgaa
	OsPYL12	F: ggaggtgaccataggctcaa
		R: gagaaaccatcgcgagtgac
	OsPYL13	F: ACCGCTATGAGCTCGTTGCAA
		R: ATCAAGGTGGATGAGTAGTC
CHIP qPCR	*AtPYL13* P1	F: GGAACATTGAATATGCCTACCCTG
		R: GTTTCTCTTAATTGGTTGCCGACC
	AtPYL13 P2	F: CGGGCCTTATTTCTAATAGGCAAT
		R: GTTCTACGTATCCAATCCGAGAG
	Negative primer	F: CTAGCGTAGTCGAGACCATTG
		R: GAAGCCGGAGACTAACGTCAC
EMSA	pET28a-ABI3	F: GGACAGCAAATGGGTCGCGGATCCATGAAAAG
		R: GAGTGCGGCCGCAAGCTTGTCGACTCATTTAACAGTTTGAG
	PYL13Pro-WT-T7	F: ACTCACTATAGGATGTAGAACTTGCATGGCTCTCGGATGTCATAGCTGTTT
		R: AAACAGCTATGACATCCGAGAGCCATGCAAGTTCTACATCCTATAGTGAGT
	PYL13Pro-mRY-T7	F: ACTCACTATAGGATGTAGAACTcaggatGCTCTCGGATGTCATAGCTGTTT
		R: AAACAGCTATGACATCCGAGAGCatcctgAGTTCTACATCCTATAGTGAGT
	CY5 tagged T7	F: TAATACGACTCACTATAGG
Y1H	ADABI3-B3	F: gccatggaggccagtgaattcTTGCAGAAAGTCTTGAAGCAAAGC

		R: agctcgagctcgatggatccTCATCTTACTTTAACCCCTCGTATCA
	ADABI3-KKS-B3	F: aaatacaagttttggtccAACAACAAAAGCAGGATGTATCTCC
		R: ggaccaaaacttgtatttCATGTTCCAAACACGAGAGGTTC
	13-Y1H	F: cGAACTTGCATGGCTCTGAACTTGCATGGCTCTGAACTTGCATGGCTCTg
		R: tcgacAGAGCCATGCAAGTTCAGAGCCATGCAAGTTCAGAGCCATGCAAGTTCggtac
	m13-Y1H	F: cGAACTaaaaaaGCTCTGAACTaaaaaaGCTCTGAACTaaaaaaGCTCTg
		R: tcgacAGAGCttttttAGTTCAGAGCttttttAGTTCAGAGCttttttAGTTCggtac
GUS reporter line	*PYL13Pro*(-2000) Vector: pCAMBIA1381	F: TGTTGGGCCCGGCGCGCCGAATTCAGTATACTATATGTTATTCATGAGT
		R: CTTAAAGCTTGGCTGCAGGTCGACTTTCTGTTTTGTTTTTCTAATGGG
Y2H	Paraburk-BD	F: atggccatggaggccgaattcATGACAGTTGTGGTGACCG
		R: ggccgctgcaggtcgacggatccTCATCCAACACTTCTAAGAG
	Frankia-BD	F: atggccatggaggccgaattcATGGATGTTGAGGTGGATG
		R: ggccgctgcaggtcgacggatccTCAAGAAGCAGATCCACTAAC
	Pseudo-BD	F: atggccatggaggccgaattcATGAAGGCTACCATTACC
		R: ggccgctgcaggtcgacggatccTCAAGAAGCAAGATTCAAAG
	Sphingomo-BD	F: atggccatggaggccgaattcATGAGATCAGAAGGAGAAG
		R: ggccgctgcaggtcgacggatccTCAAGGTGCTCCAAGACCC
LCI	nLUC-MP3	F: ACGAGCTCGGTACCCGGGATCCATGGGTTCTTTGGTCAGCc
		R: GACGCGTACGAGATCTGGTCGACATTGGGCGTGAGCGaaa
	nLUC-PrPYL	F: gctcggtacccgggatccATGACAGTTGTGGTGAC
		R: gtacgagatctggtcgacTCCAACACTTCTAAGAGCAC
in vitro phosphorylation	28a-MeenPYL3	F: GGACAGCAAATGGGTCGCGGATCCATGGGTTCTTTGGTCAGCCA
		R: GAGTGCGGCCGCAAGCTTGTCGACATTGGGCGTGAGCGAAA
	28a-PrPYL	F: ACAGCAAATGGGTCGCGGATCCATGACAGTTGTGGTGACCG
		R: GTGCGGCCGCAAGCTTGTCGACTCATCCAACACTTCTAAGAG
RT-PCR for transgenic line	*PYL1-HA*	PYL1-F: ATGGCGAATTCAGAGTCCTCC
		HA-R: gtcatacggatagcccgcatag
	ACTIN3	F: CCTCATGCCATCCTCCGTCT
		R: CAGCGATACCTGAGAACATAGTGG

Supplementary References

1. Fujii, H. *et al. In vitro* reconstitution of an abscisic acid signalling pathway. *Nature* **462**, 660-4 (2009).
2. Zhao, Y. *et al. Arabidopsis* duodecuple mutant of PYL ABA receptors reveals PYL repression of ABA-independent SnRK2 activity. *Cell Rep* **23**, 3340-3351 (2018).
3. Wang, P. *et al.* Reciprocal regulation of the TOR kinase and ABA receptor balances plant growth and stress response. *Mol Cell* **69**, 100-112 e6 (2018).
