# Supplementary material for: ABA-independent PP2C-binding in PYLs traces to bacterial origins and persists in land plants
Source: Nat Commun. 2025 Dec 16;16:11261. doi: 10.1038/s41467-025-66141-9 (PMC12717121; doi:10.1038/s41467-025-66141-9)
Supplement: Supplementary file 2 — Description of Additional Supplementary Files [file 41467_2025_66141_MOESM2_ESM.pdf]

Description of Additional Supplementary Files

Supplementary Data 1: Phylogenetic analysis of PYLs from the representative land plants and streptophyte algae.

Phylogenetic analysis of PYLs from the genome and transcriptome data of representative land plants and streptophyte algae. The two critical ABA-binding residues are listed. A third residue represents a conserved serine residue that also determines the ABA responsiveness of PYLs3.
