# Supplementary figures and images for: ABA-independent PP2C-binding in PYLs traces to bacterial origins and persists in land plants
Source: Nat Commun. 2025 Dec 16;16:11261. doi: 10.1038/s41467-025-66141-9 (PMC12717121; doi:10.1038/s41467-025-66141-9)

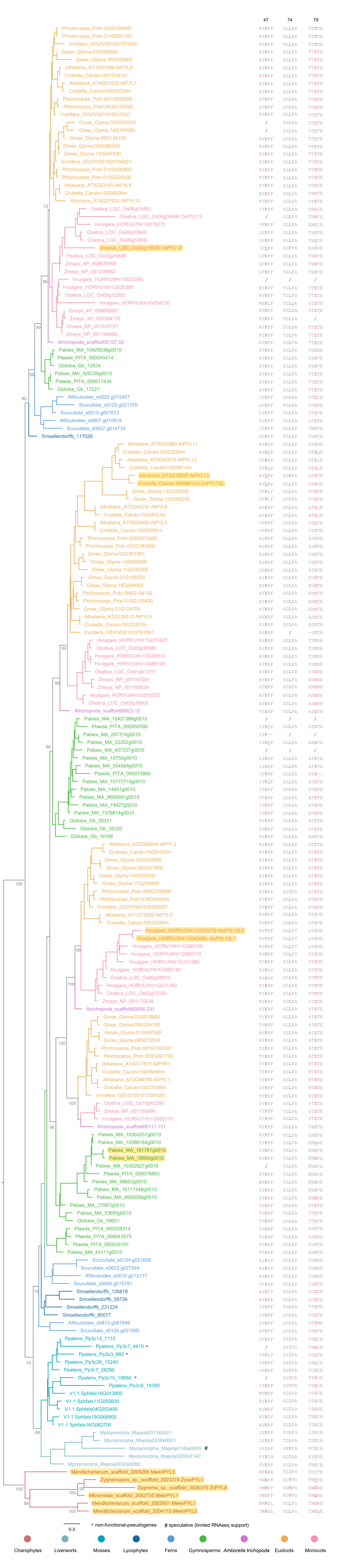

Supplement: Supplementary file 3 — Supplementary Data 1 [file 41467_2025_66141_MOESM3_ESM.pdf]
