# Supplementary material for: ABA-independent PP2C-binding in PYLs traces to bacterial origins and persists in land plants
Source: Nat Commun. 2025 Dec 16;16:11261. doi: 10.1038/s41467-025-66141-9 (PMC12717121; doi:10.1038/s41467-025-66141-9)
Supplement: Supplementary file 4 — Reporting Summary [file 41467_2025_66141_MOESM4_ESM.pdf]

nature portfolio

Corresponding author(s): Yang Zhao

Last updated by author(s): Oct 28, 2025

Reporting Summary

Nature Portfolio wishes to improve the reproducibility of the work that we publish. This form provides structure for consistency and transparency in reporting. For further information on Nature Portfolio policies, see our Editorial Policies and the Editorial Policy Checklist.

Statistics

For all statistical analyses, confirm that the following items are present in the figure legend, table legend, main text, or Methods section.

n/a	Confirmed	
☐	☒	The exact sample size (*n*) for each experimental group/condition, given as a discrete number and unit of measurement
☐	☒	A statement on whether measurements were taken from distinct samples or whether the same sample was measured repeatedly
☐	☒	The statistical test(s) used AND whether they are one- or two-sided<br>*Only common tests should be described solely by name; describe more complex techniques in the Methods section.*
☒	☐	A description of all covariates tested
☒	☐	A description of any assumptions or corrections, such as tests of normality and adjustment for multiple comparisons
☐	☒	A full description of the statistical parameters including central tendency (e.g. means) or other basic estimates (e.g. regression coefficient) AND variation (e.g. standard deviation) or associated estimates of uncertainty (e.g. confidence intervals)
☐	☒	For null hypothesis testing, the test statistic (e.g. *F*, *t*, *r*) with confidence intervals, effect sizes, degrees of freedom and *P* value noted<br>*Give P values as exact values whenever suitable.*
☒	☐	For Bayesian analysis, information on the choice of priors and Markov chain Monte Carlo settings
☒	☐	For hierarchical and complex designs, identification of the appropriate level for tests and full reporting of outcomes
☒	☐	Estimates of effect sizes (e.g. Cohen's *d*, Pearson's *r*), indicating how they were calculated

Our web collection on statistics for biologists contains articles on many of the points above.

Software and code

Policy information about availability of computer code

Data collection	qPCR: CFX Connect Real-Time PCR Detection System.<br>In vitro kinase assay: Amersham Typhoon scanner.<br>Firefly luciferase complementation imaging (LCI) assay: CCD Tanon-5200.<br>PYL-mediated PP2C enzyme assay: spectraMax-i3x.<br>Imaging acquisition of GUS staining results in seed: OLYMPUS SZX7.
Data analysis	Graph generation and statistical analyses: GraphPad Prism 7 and Microsoft Excel (v16.32).<br>Multiple sequence alignment and phylogenetic analysis: MEGA12 and ClustalW.<br>Protein structure prediction: alphafold3 (https://alphafoldserver.com).

For manuscripts utilizing custom algorithms or software that are central to the research but not yet described in published literature, software must be made available to editors and reviewers. We strongly encourage code deposition in a community repository (e.g. GitHub). See the Nature Portfolio guidelines for submitting code & software for further information.

Data

Policy information about availability of data

All manuscripts must include a data availability statement. This statement should provide the following information, where applicable:

- Accession codes, unique identifiers, or web links for publicly available datasets
- A description of any restrictions on data availability
- For clinical datasets or third party data, please ensure that the statement adheres to our policy

All data supporting the findings of this study are available in the main text or the supplementary files. The biological materials are available from the corresponding author upon reasonable request. Source data are provided with this paper. Genes mentioned in this study can be found in the TAIR database (https://www.arabidopsis.org/) or NCBI Database (https://www.ncbi.nlm.nih.gov/) under the following accession numbers: AlPYRL8, XP_020877794, A. lyrata; AlPYRL11, XP_002870032, A. lyrata; CaPYL13L1, PHT90407, Capsicum annuum; CaPYL13L2, XP_016558326, Capsicum annuum; CbPYL13L, PHT30423, Capsicum baccatum; CrPYL13L, XP_006285406, Capsella rubella; CsPYL13L1, XP_010449490, Camelina sativa (false flax); CsPYL13L2, XP_010434508, Camelina sativa (false flax); HvPYL13L1, HORVU3Hr1G040680.1, Hordeum vulgare L.; HvPYL13L2, HORVU3Hr1G030210.4, Hordeum vulgare L.; NaPYL13L, XP_019234962, Nicotiana attenuata; NsPYL13L, XP_009802216, Nicotiana sylvestris; NtPYL13L, XP_009595006, Nicotiana tomentosiformis; ObPYL12, XP_006648510.1, Oryza brachyantha; OsPYL12, XP_015624089, Oryza sativa.

Research involving human participants, their data, or biological material

Policy information about studies with human participants or human data. See also policy information about sex, gender (identity/presentation), and sexual orientation and race, ethnicity and racism.

Reporting on sex and gender	n/a
Reporting on race, ethnicity, or other socially relevant groupings	n/a
Population characteristics	n/a
Recruitment	n/a
Ethics oversight	n/a

Note that full information on the approval of the study protocol must also be provided in the manuscript.

Field-specific reporting

Please select the one below that is the best fit for your research. If you are not sure, read the appropriate sections before making your selection.

☒ Life sciences ☐ Behavioural & social sciences ☐ Ecological, evolutionary & environmental sciences

For a reference copy of the document with all sections, see nature.com/documents/nr-reporting-summary-flat.pdf

Life sciences study design

All studies must disclose on these points even when the disclosure is negative.

Sample size	For the relative gray intensity of the band in the in vitro kinasy assay, sample sizes were n = 3 (except Fig. 1E and 1F: n = 2); for PYL-mediated PP2C enzyme assay, n = 3; for transient expression in protoplasts, n ≥ 3; for firefly luciferase complementation imaging (LCI) assay, n ≥ 3; for CHIP-qPCR, n ≥ 3.
Data exclusions	No data exclusion.
Replication	For in vitro kinase assay in Fig 1E and 1F, two independent biological replicates were performed. For CHIP-qPCR in Fig 5G, two independent biological replicates were performed. For other experiments, a minimum of three independent biological replicates was performed.
Randomization	Plant pot and plate positioning in the growth chambers were randomized to minimize positional effects during growth.
Blinding	No blinding was used since measurements were not vulnerable to observer bias. Data were always collected according to the genotype of plants.

Reporting for specific materials, systems and methods

We require information from authors about some types of materials, experimental systems and methods used in many studies. Here, indicate whether each material, system or method listed is relevant to your study. If you are not sure if a list item applies to your research, read the appropriate section before selecting a response.

Materials & experimental systems

n/a	Involved in the study	
[ ]	[x]	Antibodies
[x]	[ ]	Eukaryotic cell lines
[x]	[ ]	Palaeontology and archaeology
[x]	[ ]	Animals and other organisms
[x]	[ ]	Clinical data
[x]	[ ]	Dual use research of concern
[ ]	[x]	Plants

Methods

n/a	Involved in the study	
[x]	[ ]	ChIP-seq
[x]	[ ]	Flow cytometry
[x]	[ ]	MRI-based neuroimaging

Antibodies

Antibodies used

Primary antibodies:
Anti-Actin antibody (ABclonal, AC009), 1:5000;
Anti-MYC antibody (ABclonal, AE010), 1:5000;
Anti-MBP antibody (Abmart, M20051M), 1:5000;
Anti-HIS antidody (ABclonal, AE086), 1:5000;
Anti-phospho-S175-SnRK2.2/3/6 antibody (ABclonal, AP1481), 1:3000.
Secondary antibodies:
Anti-Rabbit HRP-conjugated antibodies (BioRad, 1721019), 1:10000;
Anti-Mouse HRP-conjugated antibodies (BioRad, 1721011), 1:10000.

Validation

Anti-Actin antibody (ABclonal, AC009): Actin (plant specific) Mouse mAb. The validation of this antibody was described on the manufacturer's website (https://abclonal.com.cn/catalog/AC009).
Anti-MYC antibody (ABclonal, AE010): Mouse anti Myc-Tag mAb. The validation of this antibody was described on the manufacturer's website (https://abclonal.com.cn/catalog/AE010).
Anti-MBP antibody (Abmart, M20051M): MBP-Tag(4M14) Mouse mAb. The validation of this antibody was described on the manufacturer's website (https://www.ab-mart.com.cn/page.aspx?node=%2059%20&id=%2017734).
Anti-HIS antidody (ABclonal, AE086): Rabbit anti His-tag mAb. The validation of this antibody was described on the manufacturer's website (https://abclonal.com.cn/catalog/AE086).
Anti-phospho-S175-SnRK2s antibody (ABclonal, AP1481): Phospho-SnRK2.6-S175 pAb. The validation of this antibody was described on the manufacturer's website (https://abclonal.com.cn/catalog/AP1481).

Dual use research of concern

Policy information about dual use research of concern

Hazards

Could the accidental, deliberate or reckless misuse of agents or technologies generated in the work, or the application of information presented in the manuscript, pose a threat to:

No	Yes	
[x]	[ ]	Public health
[x]	[ ]	National security
[x]	[ ]	Crops and/or livestock
[x]	[ ]	Ecosystems
[x]	[ ]	Any other significant area

Experiments of concern

Does the work involve any of these experiments of concern:

No	Yes	
☒	☐	Demonstrate how to render a vaccine ineffective
☒	☐	Confer resistance to therapeutically useful antibiotics or antiviral agents
☒	☐	Enhance the virulence of a pathogen or render a nonpathogen virulent
☒	☐	Increase transmissibility of a pathogen
☒	☐	Alter the host range of a pathogen
☒	☐	Enable evasion of diagnostic/detection modalities
☒	☐	Enable the weaponization of a biological agent or toxin
☒	☐	Any other potentially harmful combination of experiments and agents

Plants

Seed stocks	Seeds stocks have been described in detail in the manuscript.
Novel plant genotypes	The generation of new plant genotypes has been described in detail in the manuscript methods.
Authentication	The manuscript describes in detail all the identification methods used to identify the (novel) genotypes in this study.
